# Inhibition of stromal biglycan promotes normalization of the tumor microenvironment and enhances chemotherapeutic efficacy

**DOI:** 10.1186/s13058-021-01423-w

**Published:** 2021-05-10

**Authors:** Li Cong, Nako Maishi, Dorcas A. Annan, Marian F. Young, Hirofumi Morimoto, Masahiro Morimoto, Jin-Min Nam, Yasuhiro Hida, Kyoko Hida

**Affiliations:** 1 Vascular Biology and Molecular Pathology, Graduate School of Dental Medicine, Hokkaido University, Sapporo, 060-8586 Japan; 2 Vascular Biology, Frontier Research Unit, Institute for Genetic Medicine, Hokkaido University, Sapporo, 060-0815 Japan; 3 Molecular Biology of Bones and Teeth Section, NIDCR, Bethesda, MD 20892-4320 USA; 4 Global Institution for Collaborative Research and Education (GI-CoRE), Faculty of Medicine, Hokkaido University, Sapporo, 060-0808 Japan; 5 Department of Cardiovascular and Thoracic Surgery, Faculty of Medicine, Hokkaido University, Sapporo, 060-8638 Japan

**Keywords:** Tumor stroma, Angiogenesis, Biglycan, Tumor microenvironment, Breast cancer

## Abstract

**Background:**

Biglycan is a proteoglycan found in the extracellular matrix. We have previously shown that biglycan is secreted from tumor endothelial cells and induces tumor angiogenesis and metastasis. However, the function of stroma biglycan in breast cancer is still unclear.

**Methods:**

Biglycan gene analysis and its prognostic values in human breast cancers were based on TCGA data. E0771 breast cancer cells were injected into WT and *Bgn* KO mice, respectively.

**Results:**

Breast cancer patients with high biglycan expression had worse distant metastasis-free survival. Furthermore, biglycan expression was higher in the tumor stromal compartment compared to the epithelial compartment. Knockout of biglycan in the stroma (*Bgn* KO) in E0771 tumor-bearing mice inhibited metastasis to the lung. *Bgn* KO also impaired tumor angiogenesis and normalized tumor vasculature by repressing tumor necrosis factor-ɑ/angiopoietin 2 signaling. Moreover, fibrosis was suppressed and CD8+ T cell infiltration was increased in tumor-bearing *Bgn* KO mice. Furthermore, chemotherapy drug delivery and efficacy were improved in vivo in *Bgn* KO mice.

**Conclusion:**

Our results suggest that targeting stromal biglycan may yield a potent and superior anticancer effect in breast cancer.

## Background

Tumor vasculature is structurally abnormal relative to normal vasculature and is characterized by highly permeable and leaky blood vessels [1]. The abnormality of tumor vasculature impairs blood perfusion and oxygenation, resulting in hypoxia and acidosis, and thus promoting tumor growth and progression. Furthermore, the leakiness of tumor blood vessels results in spontaneous hemorrhages and elevated interstitial fluid pressure (IFP) [2]. Hypoperfusion, hypoxia, and high IFP impede the functions of immune cells in tumors, in addition to impeding the transport of therapeutic agents [3]. As a result, abnormal tumor vasculature can promote drug resistance and hinder anticancer activity.

Antiangiogenic therapy has been considered as a promising treatment strategy for breast cancer [4]. Bevacizumab, a neutralizing antibody against vascular endothelial growth factor (VEGF), mediates antitumor effects by blocking tumor blood supply [5]. Furthermore, increased pericyte recruitment and vessel functions using bevacizumab has been shown in breast cancer patients [6, 7]. Bevacizumab received FDA approval for metastatic breast cancer in 2008. However, other trials found no significant effect on prolonging progression-free survival using Bevacizumab resulting in withdrawal of FDA approval [8, 9]. Thus, alternative strategies to modulate abnormal tumor vasculature in breast cancer are still needed.

Tumor endothelial cells (TECs) differ from normal endothelial cells (NECs) in several aspects, such as gene expression profiles [10], proangiogenic properties [11], sensitivity to drugs [12], and responses to growth factors [13]. Furthermore, TECs are cytogenetically abnormal [14]. By comparing the gene expression profiles of TECs and NECs, we have previously shown that biglycan gene expression levels are significantly elevated in isolated TECs [15].

Biglycan is a small leucine-rich proteoglycan (SLRP), consisting of a 42-kDa core protein and chondroitin sulfate/dermatan sulfate side chains [16]. Biglycan is involved in the migration of lung fibroblasts, the inflammatory response in renal macrophages, and abnormal collagen fibril morphology in bones [17–19]. Under physiological conditions, biglycan is sequestered in the extracellular matrix (ECM). Biglycan is proteolytically cleaved from the ECM upon tissue stress or injury in a soluble form, thereby acting as a damage-associated molecular pattern (DAMP) protein [20]. Biglycan secreted from TECs can also induce proangiogenic effects in an autocrine manner [15] and has been shown to promote tumor cell intravasation and metastasis through the NF-κB and ERK signaling pathways [21].

Biglycan has also been detected in several types of human cancers cells, such as endometrial cancer, bladder cancer, and pancreatic adenocarcinoma [22–24]. Functionally, biglycan expressed in cancer cells is involved in tumor growth and progression, depending on tumor type. However, the role of biglycan in tumor stroma has not been clarified. In the present study, we focused on the functional role of stromal biglycan in breast cancer microenvironment using biglycan knockout (*Bgn* KO) mice. Additionally, we investigated the therapeutic efficacy of targeting stromal biglycan combined with conventional chemotherapy in breast cancer.

## Materials and methods

### Bioinformatic analysis

We compared biglycan mRNA levels of human breast cancers, normal mammary gland, and tumor stromal and epithelial parts using Oncomine (https://www.oncomine.com/). The prognostic significances of biglycan mRNA and protein levels in human breast cancers were evaluated using the Kaplan-Meier plotter (http://kmplot.com/analysis/). Co-expression of biglycan and angiogenesis-related genes was determined using the cBioPortal database (https://www.cbioportal.org/).

### Cell lines and culture conditions

MS1 cells (mouse immortalized islet-derived normal endothelial cells) were obtained from American Type Culture Collection (Manassas, VA). MS1 cells were cultured in Dulbecco’s minimum essential medium (DMEM, Sigma-Aldrich, St. Louis, MO, USA) supplemented with 10% heat-inactivated fetal bovine serum (FBS) and 1% penicillin/streptomycin antibiotics (Sigma-Aldrich). E0771 cells were purchased from CH3 BioSystems and cultured in RPMI 1640 medium (Sigma-Aldrich) supplemented with 10 mM HEPES and 10% FBS. RAW264.7 cells were obtained from DS Pharma Biomedical and cultured in DMEM with 10% FBS. Dermal endothelial cells from C57BL/6 mice were purchased from Cell Biologics and cultured in EGM-2MV medium (Lonza, Basel, Switzerland). NIH3T3 cells from ATCC were cultured in DMEM with 10% FBS. All cells were cultured at 37 °C in a humidified atmosphere containing 5% CO_2_. The absence of *Mycoplasma pulmonis* was verified by polymerase chain reaction (PCR).

### Mouse tumor xenograft model and drug administration

All animal experiments were approved by the animal research authorities of Hokkaido University. Wild type (WT) C57BL/6 mice (female, 7–8 weeks old, 17–20 g) were purchased from CLEA Japan (Tokyo, Japan). *Bgn* KO C57BL/6 mice (female, 7–9 weeks old, 18–20 g) were used in this study. *Bgn* KO C57BL/6 mice were established by Marian F. Young (National Institutes of Health, USA) [25]. Briefly, Bgn-deficient mice were originally generated by using homologous recombination in embryonic stem cells. Biglycan was disrupted by inserting the PGK-neo cassette from the pPNT vector into exon 2 at the Tth111I site. The genotype of Bgn KO mice was determined by a PCR-based assay using the combination of a primer containing the PGK promoter sequences (5′-tggatgtggaatgtgtgcgagg-3′) of the targeted allele along with a forward primer corresponding to the 5′-end of exon 2 (5′-caggaacattgaccatg-3′) and a reverse primer corresponding to the 3′-end of exon 2 (5′-gaaaggacacatggcactgaag-3′). Biglycan was disrupted throughout the whole cells of the body. E0771 cells were transfected with lentiviral vectors encoding the tdTomato and luciferase genes as previously described [21]. tdTomato/luc2-expressing E0771 cells (1 × 10^6^ cells) suspended in an equal volume of Hank’s balanced salt solution (HBSS) and Matrigel (Corning, 356231) were orthotopically implanted into the left fourth mammary gland of WT and Bgn KO mice (5 mice per group). Tumor volumes were measured with a caliper every 3 or 4 days and calculated using the formula: volume (V) = largest dimension × smallest dimension^2^ × 0.5. For histological analysis of tumors, mice were sacrificed 24 days after implantation. Lungs were analyzed for tumor metastasis by ex vivo bioluminescence imaging using the IVIS Spectrum system (Caliper Life Science). To evaluate the effect of *Bgn* KO on chemotherapy, mice were randomly assigned to 4 groups (4 or 5 mice per group) after tumor formation (7 days after tumor cell inoculation), and treated with paclitaxel (Taxol, EnZo, BML0T104) (2 mg/kg) or DMSO as a control via intraperitoneal (i.p.) injection every 3 days, for a total of 6 injections. After 22 days, mice were sacrificed via cervical dislocation after isoflurane anesthesia. Tumor tissues, lungs, and left inguinal lymph nodes were dissected. The IVIS imaging system was used to detect metastasis in lungs and lymph nodes.

### Isolation of TECs

TECs derived from E0771 tumors were isolated as previously described, with some modifications [14]. Briefly, E0771 tumors grown in C57BL/6 mice (*n* = 10) were minced. TECs were sorted using a magnetic cell sorting system (Miltenyi Biotec, Tokyo, Japan) with anti-mouse CD31 microbeads according to the manufacturer’s instructions. To improve the purity of TECs, CD45−/CD31+ cells were sorted using a FACS Aria II (BD Biosciences). Isolated cells were maintained in EGM-2MV medium (Lonza, Basel, Switzerland) with 15% FBS. All purified ECs were cultured in EGM-2MV and were used between passages 12 and 20.

### Histological analysis

Tumor tissues were dissected from mice and processed for embedding in paraffin or in OCT compound. Paraffin sections (4 μm) were stained with anti-mouse CD31 antibody (Abcam, ab28364) and counterstained with hematoxylin. Frozen tissue samples were cut into 10-μm sections using a cryostat (Leica CM3050S, Leica Biosystems, Wetxlar, Germany). Sections were fixed in 100% ice-cold acetone for 30 min and permeabilized with 0.1% Triton in PBS at room temperature (RT) for 15 min. Sections were then blocked in PBS containing 5% bovine serum albumin (BSA) or 5% goat serum and 0.05% Triton X-100. Frozen tumor tissues were double stained using Alexa Fluor 647-conjugated anti-mouse CD31 antibody (Biolegend, 102416) and anti-biglycan (Kerafast, LF-159) antibodies to determine the co-localization of CD31 and biglycan in ECs. Pericyte-covered tumor blood vessels in frozen sections were visualized by the co-staining of rat anti-mouse CD31 (BD Pharmingen, 553370) and rabbit anti-mouse α-SMA (Abcam, ab5694) followed by Alexa Fluor 647-conjugated goat anti-rat IgG (Biolegend, 405416) and Alexa Fluor 488-conjugated goat anti-rabbit IgG (Invitrogen, 11034) for 2 h at room temperature. To detect hypoxia in tumors, frozen sections were stained with an anti-glucose transporter Glut1 antibody (Abcam, ab115730) followed by Alexa Fluor 647-conjugated goat anti-rabbit secondary antibody (Invitrogen, 21244). CD8+ T cells in frozen sections were stained by rat anti-mouse CD8a (Biolegend, 100702) followed by Alexa Fluor 647-conjugated goat anti-rat IgG (Biolegend, 405416). Frozen sections were counterstained with 4,6-diamidino-2-phenylindole (DAPI; Dojin), and images were obtained using a BZ-X810 microscope equipped with BZ-X800 Analyzer software (Keyence Corporation, Itasca, IL, USA). The area of CD31+ or Glut1+ staining was quantified using ImageJ software (NIH), and the percentage of CD31+ or Glut1+ area was calculated as a percentage of the positive area to the total area. The area of pericyte-covered tumor blood vessels was calculated by the ratio of the number of blood vessels co-stained with α-SMA and CD31 to the total number of CD31-positive blood vessels. Collagen accumulation was evaluated by Picro Sirius Red Stain Kit (Abcam, ab150681). Five fields per tumor were quantified and values were averaged to obtain one value for each tumor. Each group consisted of 4 or 5 mice.

### Assessment of perfused blood vessels

Doxorubicin (15 mg/kg, Sigma) was injected into the tail vein of E0771 tumor-bearing mice. FITC-lectin (2 mg/kg, Vector Laboratories) was intravenously injected via tail vein 4 h after doxorubicin injection. Tumors were extracted 5 min after injection and frozen in blocks. These frozen sections were stained with Alexa Fluor 488-conjugated anti-mouse CD31 antibody (Biolegend, 12514) or Alexa Fluor 647-conjugated anti-mouse CD31 antibody (Biolegend, 102416). The area of lectin- or doxorubicin-positive staining was determined using ImageJ software.

### RNA isolation, reverse transcription PCR, and quantitative real-time PCR

RNA from cells and tissues was isolated using the ReliaPrep RNA tissues miniprep system (Promega) and the RNeasy Micro kit (Qiagen), respectively. cDNA, reverse transcription, PCR, and quantitative real-time PCR were performed as previously described [26]. The primers used in this study are listed in Supplementary Table S1.

### ELISA

Biglycan concentration in mouse plasma was determined using an ELISA kit for mouse biglycan (Cloud-clone Corp. SEJ226Mu). TNF-ɑ concentration in mouse plasma was determined using a Mouse TNF-ɑ Quantikine ELISA kit (R&D systems, MTA00B) according to the manufacturer’s instructions. Absorbance was detected using a microplate reader (Promega GloMax Multi Detection System, Promega) at 450 nm.

### Stimulation of cells with recombinant protein

E0771 cells were cultured in serum-free medium for 24 h before treatment with 5 μg/ml recombinant biglycan (Genway, GWB-ATG116) in serum-free RPMI medium. After incubation for 6 h with recombinant biglycan protein, RNA was isolated. E0771 cells were pretreated with anti-TLR2 (1 μg/ml, Biolegend, #246294) or TLR4 (10 μg/ml, Biolegend, #117608) antibody for 2 h. E0771 cells were then treated with serum-free RPMI media containing 5 μg/ml biglycan for 6 h before RNA was extracted. E0771 cell were pretreated with NF-κB inhibitor BAY11-7082 (10 μM, Calbiochem) or MEK inhibitor U1026 (20 μM, CST) for 1 h. E0771 cells were then treated with serum-free RPMI media containing 5 g/ml biglycan for 6 h before RNA was extracted. MS1 cells were kept in serum-free medium for 24 h before treatment with 1 g/ml TNF-ɑ (Peprotech, 300-01A) in serum-free DMEM medium. After incubation for 6 h with recombinant biglycan protein, RNA was isolated. NIH3T3 cells were cultured in serum-free DMEM for 24 h before treatment with 5 μg/ml recombinant biglycan in serum-free DMEM medium. After incubation for 6 h with recombinant biglycan protein, RNA was isolated.

### Biglycan knockdown using siRNA

Biglycan siRNA (5′-UAGAGGUGCUGGAGGCCUUUFAAGU-3′, 5′ -ACUUCAAAGGCCUCCAGCACCUCUA-3′) was transfected into E0771-TECs using Lipofectamine RNAi MAX Transfection Reagent (Invitrogen, Carlsbad, CA, USA) according to the manufacturer’s instructions. Control siRNA (Invitrogen, Carlsbad, CA, USA) was used as a negative control. Knockdown efficiency was confirmed by quantitative real-time PCR.

### Cell preparation and flow cytometry

E0771 cells were stained with PE-conjugated anti-mouse CD284 (Biolegend, 117605) and Alexa Fluor 488-conjugated anti-mouse CD282 (Biolegend, 121807). To analyze CD8+ T cell infiltration in tumor tissues, E0771 tumors from WT and Bgn KO mice were dissected and digested with collagenase II and DNase I. Single-cell suspensions were stained with FITC-conjugated anti-CD45 antibody (Biolegend, 103115) and APC-conjugated anti-CD8a antibody (Biolegend, 100711). CD45 + CD8+ T cells were analyzed using a FACS Aria II. Data was analyzed using FlowJo V10 software (Tree Star Inc.).

### Statistical analysis

All data are expressed as means ± standard deviation (SD) of three independent experiments performed in triplicate. Two-sided Student’s *t* test followed by Wilcoxon rank test was used for comparison between two groups. Comparisons between multiple groups were made using one-way ANOVA, followed by a Tukey-Kramer test. One-way ANOVA and *T* tests were analyzed using IBM SPSS software. *p* values < 0.05 were considered significant.

## Results

### Biglycan is highly expressed in tumor stroma, associated with prognosis and angiogenesis-related genes in human breast cancer patients

**Fig. 1:**
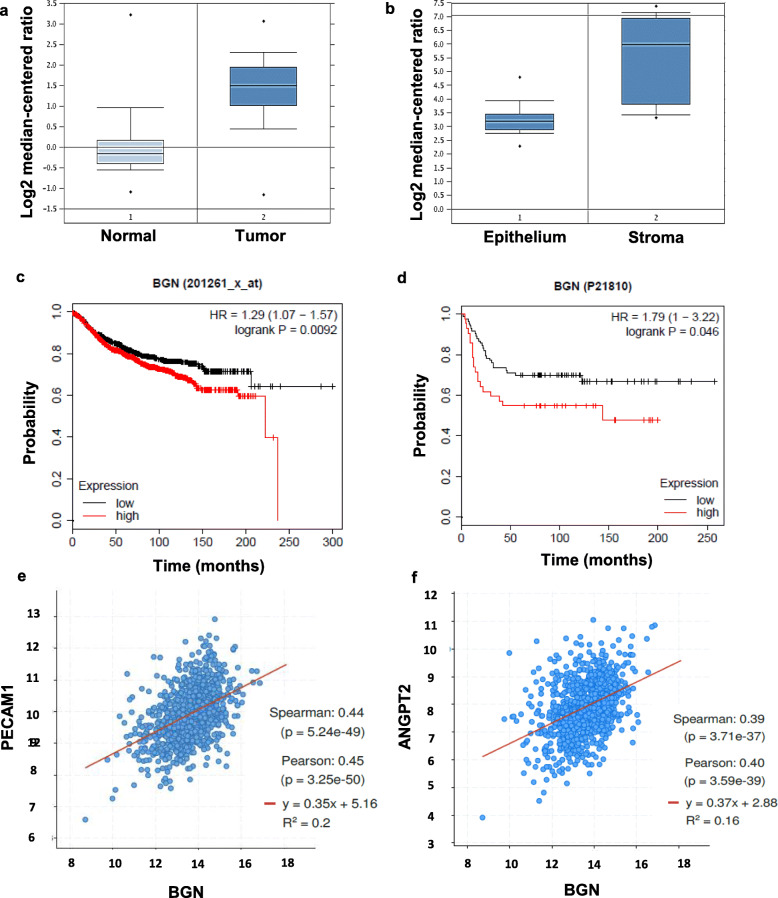
Biglycan is highly expressed in tumor stroma, associated with angiogenesis gene expression and prognosis of human breast cancer patients. **a** Comparison of biglycan mRNA expression was analyzed in normal mammary glands (*n* = 61) and breast cancer tissue (*n* = 389) using Oncomine. **b** Comparison of biglycan mRNA expression was analyzed in 14 patient-matched tumor epithelium and tumor-associated stroma specimens using Oncomine. **c** Kaplan-Meier analysis was used to assess breast cancer patients with high or low biglycan mRNA expression in distant metastasis-free survival (DMFS) using a Kaplan-Meier plotter tool. **d** Kaplan-Meier analysis was used to assess breast cancer patients with high or low biglycan protein expression in DMFS using a Kaplan-Meier plotter tool. **e** Correlation between BGN and PECAM1 expression using cBioPortal database. **f** Correlation between BGN and ANGPT2 expression using cBioPortal database

We first evaluated the expression levels of biglycan in normal mammary glands and in human breast cancer tissues using the Oncomine database (https://www.oncomine.com/). Biglycan was found to be upregulated in human breast cancers compared to normal mammary glands (Fig. 1a). Furthermore, biglycan expression was higher in the tumor stroma compartment compared to the tumor epithelial compartment of human breast cancers (Fig. 1b). The relationship between biglycan expression and prognosis of breast cancer patients was investigated using Kaplan-Meier Plotter database (http://kmplot.com/analysis/). Higher mRNA and protein expression of biglycan correlated with worse distant metastasis-free survival of breast cancer patients (Fig. 1c, d). These results suggested that biglycan is indeed upregulated in breast cancer, especially in tumor stroma, and its expression is negatively associated with the survival of breast cancer patients. Biglycan has been reported to be involved in cell migration and tube formation of ECs [15]. To determine the effect of biglycan on angiogenesis in human cancers, we analyzed the correlation between biglycan and angiogenesis-related gene expression in human breast cancers using the cBioPortal database (https://www.cbioportal.org/). Platelet endothelial cell adhesion molecule (PECAM)-1 has been shown to facilitate the interaction of endothelial cells and play a role in angiogenesis. Angiopoietin 2 (ANGPT2) destabilizes blood vessels by inducing detachment of pericytes from the endothelial cells. Pearson’s correlation coefficients showed that Biglycan mRNA expression was positively correlated with both PECAM1(Spearman’s correlation = 0.45) and ANGPT2 (Spearman’s correlation = 0.4) levels in human breast cancers (Fig. 1e, f). Taken together, these results suggest that biglycan may be involved in tumor angiogenesis and destabilization of tumor blood vessels in human breast cancers.

### Lung metastasis was decreased in *Bgn* KO mice

**Fig. 2:**
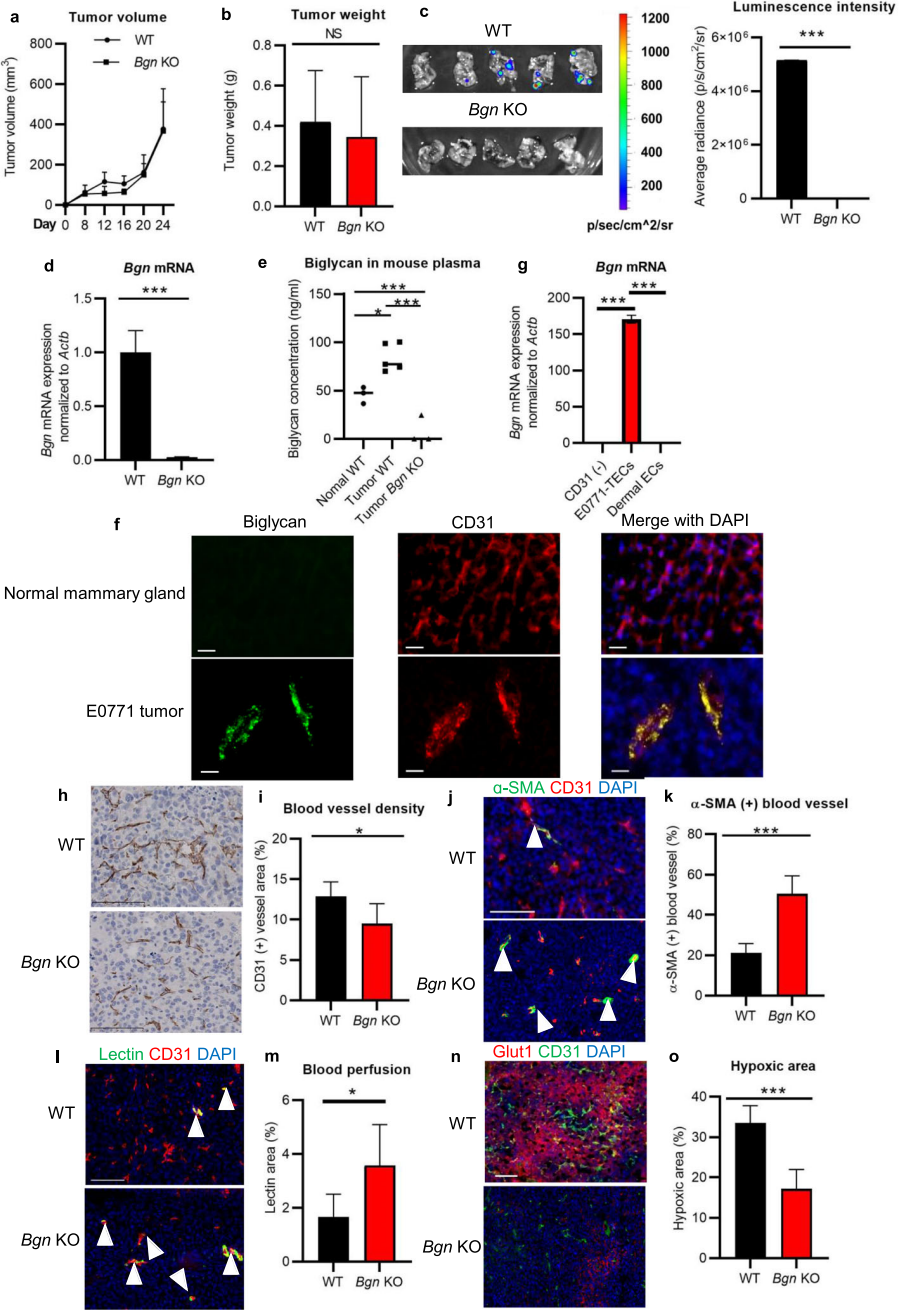
Stromal biglycan inhibition suppresses lung metastasis, by normalizing tumor vasculature in breast cancer. **a** Growth curves of E0771 tumor growth in WT and *Bgn* KO mice measured every 4 days. **b** Comparison of E0771 tumor weight at day 24 after inoculation. **c** Representative IVIS images and quantitative analysis of metastatic lungs in E0771 tumors of WT versus *Bgn* KO mice. **d** *Bgn* mRNA expression in E0771 tumor tissues of WT and Bgn KO mic analyzed by quantitative real-time RT-PCR. **e** Biglycan concentration in mouse plasma from non-tumor WT mice, E0771 tumor-bearing WT mice, and E0771 tumor-bearing *Bgn* KO mice detected by ELISA. **f** Immunofluorescent images of biglycan (green) expression in CD31+ blood vessels (red) in E0771 tumors grown in WT mice. DAPI, blue. Scale bar = 20 μm. **g** *Bgn* mRNA expression in FACS isolated CD31− cells, CD45− CD31+ cells (E0771-TECs), and dermal ECs from naïve mice by quantitative real-time RT-PCR. **h** Representative images of CD31+ blood vessels in E0771 tumors from WT and *Bgn* KO mice. Scale bar = 100 μm. **i** Quantification of blood vessel density. **j** Representative images of CD31 immunostaining (red), ɑ-SMA (green), and DAPI nuclear staining (blue) in E0771 tumors from WT and *Bgn* KO mice. Scale bar = 100 μm. **k** Quantification of ɑ-SMA+ CD31+ tumor blood vessels. The rate of microvessel pericyte coverage was analyzed by counting the vessels that stained positive for both CD31 and α-SMA (white arrowheads) among CD31-positive vessels. **l** Representative images of CD31 immunostaining (red), FITC-lectin (green), and DAPI (blue) in E0771 tumors from WT and *Bgn* KO mice. Scale bar = 100 μm. **m** Quantification of lectin+ blood vessels. The rate of lectin+ blood vessels was analyzed by counting the vessels that stained positive for both CD31 and lectin (white arrowheads) among CD31-positive vessels. **n** Representative images of CD31 immunostaining (green), Glut1 (red), and DAPI (blue) in E0771 tumors from WT and *Bgn* KO mice. Scale bar = 100 μm. **o** Quantification of Glut1+ area. *p < 0.05, ***p < 0.001. Significance in **b**, **d**, **i**, **k**, **m**, and **o** were determined by a two-tailed Student’s *t* test. Significance in **g** was determined by a one-way ANOVA. All data represent means ± SD

To investigate how stromal biglycan affects tumor growth in vivo, we orthotopically implanted murine E0771 breast carcinoma cells into the mammary fat pads of WT and *Bgn* KO female mice. *Bgn* deficiency resulted in no significant toxic effects in mice, except for a reduced growth rate and decreased bone mass [25, 27]. No significant differences were seen in primary E0771 tumor growth and tumor weight between the WT and *Bgn* KO (Fig. 2a, b). Different concentrations of recombinant biglycan also had no significant effect on proliferation of E0771 cells in vitro (Supplementary Fig. 1). However, *Bgn* KO mice showed reduced lung metastasis by IVIS (Fig. 2c). As E0771 cells had low expression of biglycan in vitro (Supplementary Fig. 2a), these results suggested that stroma biglycan is involved in lung metastasis but not in tumor growth, consistent with our previous report showing induction of metastasis by biglycan-secreting TECs [21].

We then investigated biglycan expression in the mouse E0771 tumor model. As expected, *Bgn* KO mice showed markedly reduced levels of biglycan mRNA expression in tumor tissues compared to WT mice (Fig. 2d). As biglycan is secreted in a soluble form during inflammation [17], we analyzed biglycan concentrations in the plasma of mice. Biglycan concentration was highest in tumor-bearing WT mice compared to non-tumor WT mice and tumor-bearing *Bgn* KO mice (Fig. 2e). Biglycan and CD31 were co-localized by immunofluorescence staining using CD31 and biglycan antibodies (Fig. 2f). Furthermore, to confirm the expression of biglycan in TECs, we isolated TECs from E0771 tumors in WT mice and characterized them as described previously [15]. TECs were shown to be negative for the monocyte markers CD11b and CD45, and positive for EC markers CD31, CD144, and CD105 by RT-PCR (Supplementary Fig. 2b). TECs were positive for EC markers CD31, CD105, and lectin, and negative for the hematopoietic marker CD45 by flow cytometry analysis (Supplementary Fig. 2c). These results indicate that isolated cells were highly purified ECs. We analyzed *Bgn* expression in TECs isolated from E0771 tumors (E0771-TECs), isolated CD31-negative cells including tumor cells, fibroblasts, and immune cells, and commercially available dermal ECs from normal mice (NECs) by real-time PCR. *Bgn* mRNA expression was significantly higher in E0771-TECs compared to CD31-negative cells and dermal ECs (Fig. 2g), indicating that TECs were a source of biglycan in E0771 tumor.

### Tumor angiogenesis was impaired and tumor blood vessels were normalized in *Bgn* KO mice

To determine the contribution of biglycan to angiogenesis, we first analyzed CD31+ blood vessel density in normal mammary glands from WT and *Bgn* KO mice. No significant changes were observed in morphology and density of normal mammary gland blood vessels in *Bgn* KO and WT mice (Supplementary Fig. 3a, b). Next, we evaluated the microvessel density in E0771 tumors from WT and *Bgn* KO mice. The CD31+ tumor blood vessels were significantly decreased in *Bgn* KO mice as compared to the WT (Fig. 2h, i). Additionally, the percentage of α-SMA+ pericyte-covered blood vessels was significantly higher in *Bgn* KO mice, which indicated that more mature blood vessels existed in tumors from *Bgn* KO mice compared to WT mice (Fig. 2j, k). These data suggested that tumor blood vessels in *Bgn* KO mice are structurally normalized.

Since blood vessel normalization can also enhance blood flow within vessels, we further assessed the vascular function in E0771 tumors in WT and *Bgn* KO mice. First, by intravenous lectin injection, we identified an increase in lectin-positive (functional) vessels in E0771 tumors of *Bgn* KO mice as compared to those in the WT mice, implying that intratumoral blood perfusion was enhanced in *Bgn* KO mice (Fig. 2l, m). Tumor vascular normalization is also known to elicit enhanced tumor oxygenation [3]; thus, we analyzed tumor hypoxia by staining tumor tissues with a glucose transporter (Glut1) antibody (Fig. 2n, o). Glut1+ area was decreased in tumors of *Bgn* KO mice compared to WT mice. As hypoxic response is mainly ascribed to hypoxia-inducible factor-1 (HIF-1) α which is involved in the induction of Glut1 expression [28], we checked whether biglycan affects HIF1-α and Glut1 expression in tumor cells. We found that Hif1a and Slc2a1 expression elevation in E0771 cells by stimulation with recombinant biglycan (Supplementary Fig. 4 a, b), which indicated that biglycan might mediate HIF1-α and Glut1 expression in tumor cells. Together, these data suggested that alleviating tumor hypoxia by biglycan knockout might be due to enhancement of vascular normalization and mediation of HIF1-α and Glut1 expression in tumor cells.

### Angpt2 expression was decreased in E0771 tumors from *Bgn* KO mice

**Fig. 3:**
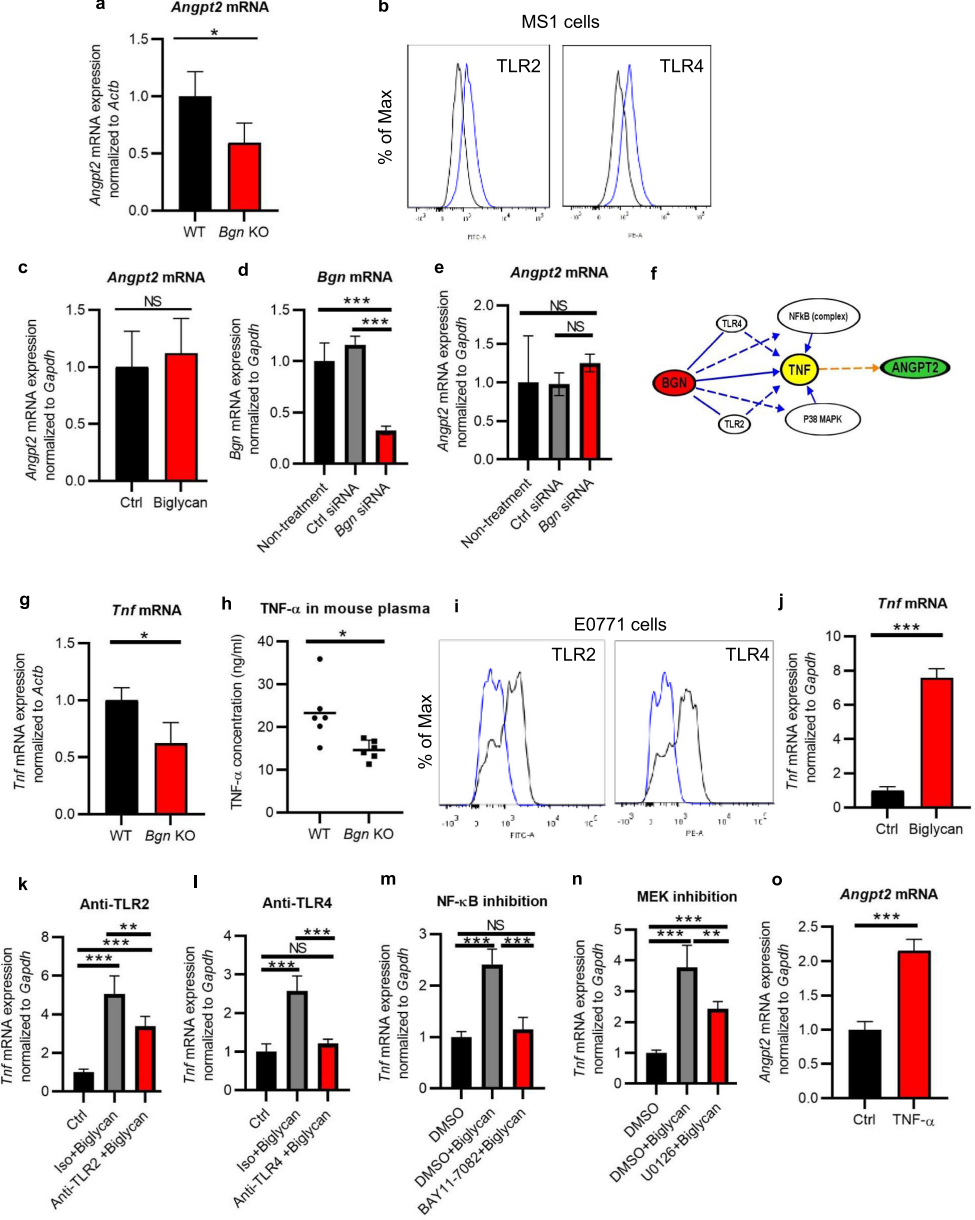
TNF-ɑ-enhanced Angpt2 expression is controlled by biglycan in E0771 tumors. **a** *Angpt2* mRNA expression in E0771 tumor tissues of WT and *Bgn* KO mice by quantitative real-time PCR. **b** TLR2 and TLR4 expression in MS1 cells analyzed by FACS analysis. Blue: antibody. Black: isotype. **c** *Angpt2* mRNA expression in MS1 cells treated with recombinant biglycan (5 g/ml) by quantitative real-time RT-PCR. **d** *Bgn* mRNA expression in E0771-TECs transfected with *Bgn* siRNA by quantitative real-time PCR. **e** *Angpt2* mRNA expression in E0771-TECs transfected with *Bgn* siRNA by quantitative real-time PCR. **f** Flowchart of biglycan, TNF-ɑ, and *Angpt2* signaling by IPA analysis. **g** *Tnf* mRNA expression by quantitative RT-PCR in E0771 tumor tissues from WT and *Bgn* KO mice. **h** TNF-ɑ secretion in the plasma of E0771 tumor-bearing mice was detected by ELISA. Each dot represents one mouse. **i** TLR2 and TLR4 expression in E0771 cells analyzed by flow cytometry. Blue: isotype. Black: antibody. **j** *Tnf* mRNA expression in E0771 cells treated with recombinant biglycan (5 μg/ml). **k, l** After blocking of TLR2 or (**k**) TLR4 (**l**), E0771 cells were stimulated by recombinant biglycan (5 μg/ml). *Tnf* mRNA expression was analyzed by quantitative RT-PCR. **m, n** After treating with (**m**) the NF-κB inhibitor (10 μM) or (**n**) the MEK inhibitor (20 μM), E0771 cells were stimulated by recombinant biglycan (5 μg/ml). *Tnf* mRNA expression was analyzed by quantitative real-time RT-PCR. **o** *Angpt2* mRNA expression in MS1 cells treated with recombinant TNF-ɑ (10 ng/ml) by quantitative RT-PCR. *p < 0.05, **p < 0.01, ***p < 0.001. NS, not significant. Significance was determined in **a**, **c**, **g**, **j**, and **o** by two-tailed Student’s *t* test, and in **d**, **e**, **k**, **l**, **m**, and **n** by a one-way ANOVA. All data represent means ± SD

Vascular normalization is regulated by several molecules, such as ANG-TIE, PDGFB, and NG2 [29]. BGN was positively correlated with ANGPT2 expression in human breast cancers (Fig. 1f). We next confirmed that *Angpt2* mRNA expression was decreased in tumor tissues from Bgn KO mice (Fig. 3a). To determine whether biglycan has any direct effect on *Angpt2* expression, we stimulated MS1 cells (immortalized normal ECs) with recombinant biglycan after confirming the expression of the biglycan receptors, Toll-like receptors (TLRs) 2 and 4 in the MS1 cells (Fig. 3b). There was no significant difference in *Angpt2* expression between biglycan-treated and non-treated cells (Fig. 3c). Furthermore, biglycan knockdown in E0771-TECs (Fig. 3d) did not alter *Angpt2* mRNA expression in the cells. (Fig. 3e). Therefore, we speculated that biglycan may not regulate *Angpt2* expression in ECs directly via its receptors, but rather by other indirect mechanisms. We further analyzed the molecular relationship between biglycan and ANGPT2 by Ingenuity Pathway Analysis (IPA, Tomy Digital Biology), and found that TNF was known to mediate this interaction (Fig. 3f). We therefore hypothesized that ANGPT2 might be regulated by biglycan/TNF-α signaling, leading to the destabilization of tumor blood vessels.

### TNF-ɑ-enhanced Angpt2 expression is controlled by biglycan through activation of NF-κB and ERK via TLR2/4

We compared *Tnf* mRNA expression and TNF-ɑ secretion in E0771 tumor-bearing WT and *Bgn* KO mice. *Bgn* KO mice showed significantly reduced *Tnf* mRNA expression in E0771 breast cancer tissues and TNF-ɑ secretion in plasma from tumor-bearing mice (Fig. 3g, h). As soluble biglycan can bind to TLRs 2 and 4 on macrophages and activate mitogen-activated protein kinase (MAPK) p38, extracellular signal-regulated kinase (ERK), and nuclear factor-κB (NF-κB) signaling pathways [30], we confirmed that E0771 cells expressed TLR2 and TLR4 by flow cytometry analysis (Fig. 3i). We proceeded to investigate the paracrine effects of biglycan on TNF-ɑ expression in E0771 cells (Fig. 3j) and RAW macrophages (Supplementary Fig. 4a) stimulated with recombinant biglycan. *Tnf* mRNA expression was significantly increased in biglycan-stimulated cells as compared to non-treated control cells (Fig. 3j). Blockage of the Bgn-TLR2 or Bgn-TLR4 interaction in E0771 cells by neutralizing antibodies significantly decreased *Tnf* mRNA expression in E0771 cells stimulated with recombinant biglycan (Fig. 3k, l), suggesting that biglycan regulates TNF-ɑ expression in tumor cells through binding to TLR2 and TLR4. To elucidate the downstream mediators of *Tnf* induction, E0771 cells were first pretreated with the NF-κB inhibitor BAY11-7082 or the ERK inhibitor U1026, and then stimulated with recombinant biglycan. NF-κB or ERK blockade decreased *Tnf* mRNA expression (Fig. 3m, n), indicating that biglycan may regulate TNF-ɑ expression through NF-κB and ERK signaling pathways.

Furthermore, we analyzed the effect of biglycan on TNF-ɑ expression in ECs. The *Tnf* mRNA expression level in MS1 cells was not changed significantly after stimulation with recombinant biglycan (Supplementary Fig. 4b). In addition, *Tnf* expression showed no difference between biglycan knockdown E0771-TECs and controls (Supplementary Fig. 4c), implying that biglycan has no autocrine effect on TNF-ɑ expression. In order to confirm that TNF-α can indeed induce *Angpt2* expression in ECs, we confirmed the presence of TNFR1 and TNFR2 expression in ECs (Supplementary Fig. 5) and stimulated MS1 cells with TNF-α. This led to increased levels of *Angpt2* mRNA in MS1 cells (Fig. 3o), consistent with previous reports. These data indicated that biglycan may regulate *Angpt2* expression via enhanced levels of TNF-α indirectly in a paracrine manner.

### Biglycan inhibition suppresses tumor fibrosis

**Fig. 4:**
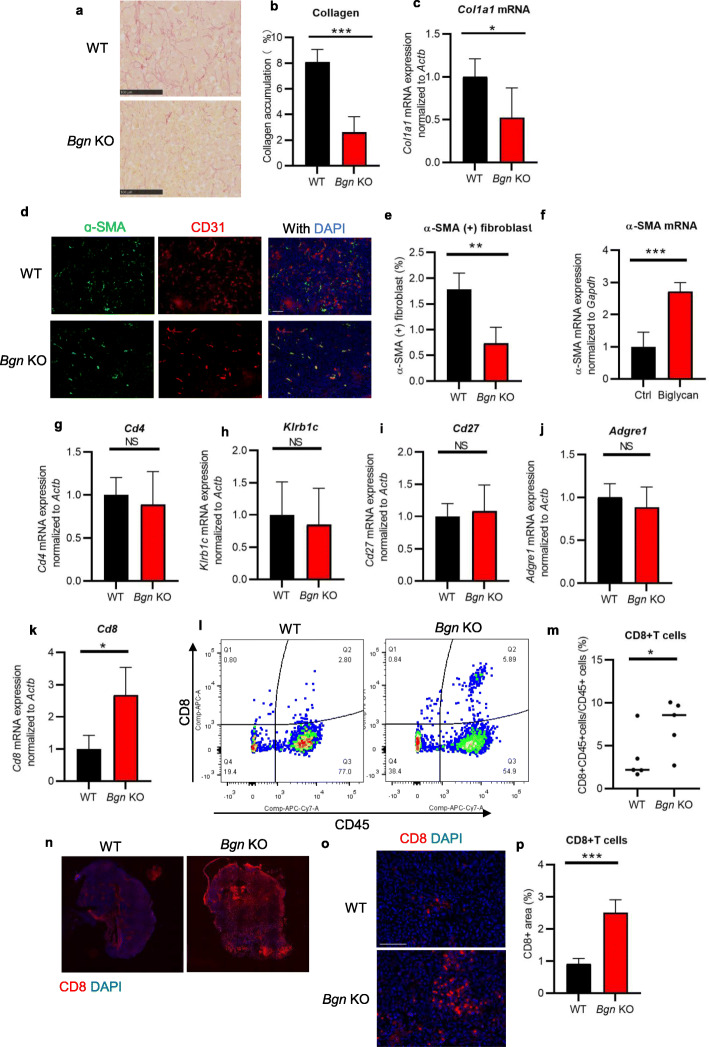
Biglycan inhibition reduces stromal fibrosis and normalizes immune suppressive microenvironment. **a** Representative images of picrosirius red staining for collagen in E0771 tumors from WT and *Bgn* KO mice. Scale bar = 100 μm. **b** Quantification of collagen accumulation. Data represent means ± SD. *n* = 5 fields, 5 mice per group. **c** *Col1a1* mRNA expression in E0771 tumor tissues of WT and *Bgn* KO mice by quantitative RT-PCR. *n* = 4 RT-PCR replicates per mouse, 5 mice per group. **d** Representative images of α-SMA (green), CD31 immunostaining (red), and DAPI nuclear staining (blue) in E0771 tumors from WT and *Bgn* KO mice. Scale bar = 100 μm.**e** Quantification of α-SMA + fibroblasts. The area of α-SMA + fibroblasts was analyzed by excluding α-SMA + pericytes from CD31+ blood vessels. **f** α-SMA mRNA expression in NIH3T3 cells treated with recombinant biglycan by quantitative real-time RT-PCR. **g** *Cd4,* **h** *Klrb1c*, **i** *Cd27*, **j** *Adgre1*, and **k** *Cd8* mRNA expression by quantitative RT-PCR in E0771 tumor tissues from WT and *Bgn* KO mice. NS, not significant. *n* = 4 RT-PCR replicates per mouse, 4–5 mice per group. **l, m** CD8 + CD45+ T cell analysis in E0771 tumors of WT and *Bgn* KO mice by flow cytometry. **l** Representative data and **m** the percentage of CD8 + CD45+ T cells/CD45+ T cells. *n* = 5 mice per group. **n**, **o** Representative images of CD8+ T cells in E0771 tumors from WT and *Bgn* KO mice. **o** in tumor center, Scale bar = 100 μm. **p** Quantification of CD8+ T cells in tumor center. *p < 0.05, **p < 0.01, ***p < 0.001. Significance was determined by two-tailed Student’s *t* test

Biglycan has been shown to interact with collagen I and functions in organization of the assembly of the extracellular matrix [31]. Furthermore, as a DAMP, biglycan potentiates renal inflammation and fibrotic renal disorders [32]. Thus, we assessed collagen deposition by picrosirius red staining in E0771 tumors. We found that collagen accumulation was reduced in tumors from *Bgn* KO mice compared to those in WT mice (Fig. 4a, b). *Col1a1* mRNA expression was also decreased in tumor tissues from *Bgn* KO mice (Fig. 4c). These findings indicate that biglycan knockout suppressed tumor fibrosis. As activated cancer-associated fibroblasts (CAFs) in tumor stroma is involved in tumor fibrosis and activated CAFs can be identified by their expression of α-SMA [33], α-SMA+ fibroblast (excluded pericytes) assessment by staining was analyzed in biglycan KO mice. α-SMA+ fibroblasts were significantly decreased in *Bgn* KO mice as compared to the WT mice (Fig. 4d, e). We also analyzed the effect of biglycan on α-SMA expression in fibroblasts and found that α-SMA mRNA expression was upregulated in NIH3T3 cells by biglycan treatment (Fig. 4f). These results indicate that biglycan may activate fibroblasts via upregulating α-SMA expression.

### Stromal biglycan deficiency increases the recruitment of CD8+ T cells in breast cancer

The vascular normalization in tumor stroma is reported to increase the accessibility of immune cells to the tumors [34]. Furthermore, a fibrotic tumor microenvironment can suppress the immune response to cancer [35]. Therefore, we analyzed the mRNA expression of several immune cell markers (*Cd4*, *Cd8*, *Klrb1c*, *CD27*, and *Adgre1*) within the E0771 tumor tissues from WT and *Bgn* KO mice (Fig. 4g–k). We found that only *Cd8a* mRNA expression was significantly increased in *Bgn* KO mice (Fig. 4k), potentially indicating an accumulation of CD8+ T cells. We confirmed this hypothesis by FACS analysis, which demonstrated that the percentage of CD45 + CD8+ T cells was higher in *Bgn* KO mice compared to WT mice (Fig. 4l, m). The infiltration of CD8+ T cells in E0771 tumors was also analyzed by IHC. Quantification of CD8+ T cells by IHC showed a significant increase in *Bgn* KO mice, with lymphocytes distributed throughout the tumor (Fig. 4n–p). Together, these data indicate that CD8+ T cell distribution in tumor tissues may be improved by Bgn inhibition.

### Biglycan inactivation enhances drug delivery and the antitumor effect of paclitaxel

**Fig. 5:**
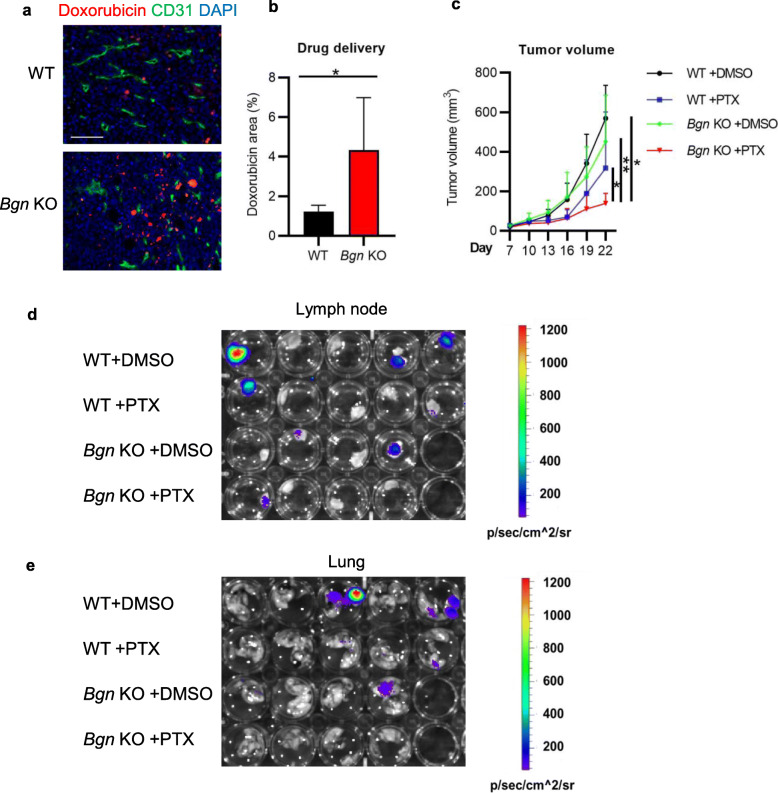
Biglycan deletion enhances drug delivery and antitumor effect of paclitaxel in breast cancer. **a** Representative images of CD31 immunostaining (green), doxorubicin (red), and DAPI nuclear staining (blue) in E0771 tumors from WT and *Bgn* KO mice. Scale bar = 100 μm. **b** Quantification of drug delivery. Significance was determined by two-tailed Student’s *t* test. *n* = 5 fields per mouse, 5 mice per group. **c** Growth curves of E0771 tumors grown in WT and *Bgn* KO mice. Mice were treated with DMSO or 2 mg/kg paclitaxel once every 3 days for a total of 6 treatments. Significance was determined by a one-way ANOVA. *n* = 4–5 mice per group. **d**, **e** Representative IVIS images of **d** metastatic lymph nodes and **e** lungs in E0771 tumors of DMSO- or paclitaxel-treated WT and *Bgn* KO mice as described in **c**. All data represent means ± SD. *p < 0.05, ***p < 0.001

Normalization of the tumor microenvironment could potentially improve drug delivery and efficiency of chemotherapy [36]. As tumor blood perfusion was improved and tumor fibrosis was inhibited in *Bgn* KO mice, we measured drug delivery and chemotherapeutic efficacy in E0771 tumors. Doxorubicin delivery was enhanced in *Bgn* KO mice (Fig. 5a, b). Additionally, the chemotherapeutic agent paclitaxel significantly suppressed E0771 tumor growth in *Bgn* KO mice (Fig. 5c). The number of lymph node metastases was decreased in paclitaxel-treated *Bgn* KO mice (1/5, 20%) as compared to paclitaxel-treated WT mice (2/5, 40%) (Fig. 5d). Paclitaxel treatment also increased the incidence of lung metastasis (2/5, 40%) in WT mice compared to *Bgn* KO mice (0/5) (Fig. 5e). These data suggested that loss of stromal biglycan enhanced chemotherapeutic efficacy in tumors via normalization of breast cancer microenvironment.

## Discussion

**Fig. 6:**
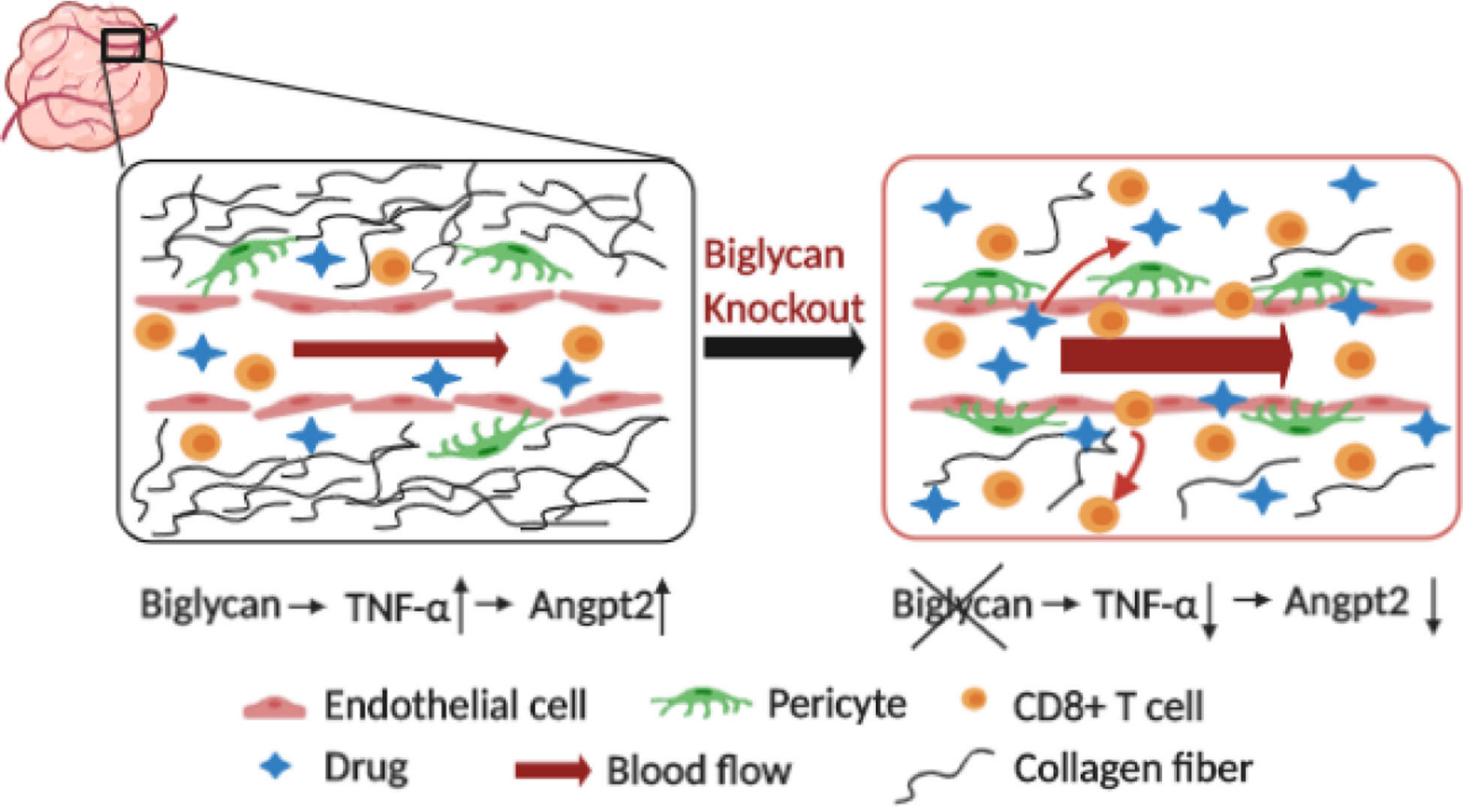
Schematic illustrating mechanisms of biglycan modulating tumor microenvironment. Inactivation of tumor stromal biglycan normalizes tumor vasculature via downregulation of *Angpt2*. Biglycan knockout in tumor stroma decreases tumor stiffness and increases the accumulation of CD8+ T cells. Thus, targeting stromal biglycan can facilitate drug delivery in tumors

Recently, the role of biglycan has been investigated in cancer cells. Biglycan has bidirectional roles modulating tumor growth and progression in different tumor types [37–39]. However, the roles of biglycan in breast cancer microenvironment remain unclear. In the current study, we found that stromal biglycan inhibition enhanced chemotherapeutic efficacy through normalization of not only the vascular but also the tumor microenvironment, resulting in increased oxygen perfusion and drug delivery (Fig. 6). To our knowledge, this is the first report demonstrating that stromal biglycan mediates destabilization of the tumor microenvironment, suggesting that biglycan can potentially serve as a therapeutic target in combination cancer therapies.

Tumor vascular normalization has been shown to enhance tumor oxygenation, reduce cancer progression, and decrease interstitial pressure in the tumor, which consequently enhances drug delivery [40]. Treatment with bevacizumab prunes some abnormal vessels and remodels the remaining vessels during the normalization window [41]. However, intrinsic and evasive drug resistance still develops, and the evaluation of the normalization window is still a challenge. There are no validated biomarkers for antiangiogenic agents to maintain normalization of tumor vasculature, and proper molecular diagnostic markers remain to be discovered [40]. In the current study, high expression of stromal biglycan associated with a worse outcome in human breast cancers, indicating that tumor stromal biglycan might play a role in cancer progression.

Biglycan has been reported to be expressed in the tumor microenvironment [15, 21, 42]. We previously showed that biglycan is highly expressed in TECs and facilitates cancer metastasis [15, 21]. In our study, biglycan was only expressed in tumor blood vessels of mouse breast cancers, and not in normal mammary gland tissue. Furthermore, co-expression analysis by cBioPortal showed that BGN was positively associated with PECAM1 and ANGPT2 expression in human breast cancers. Both PECAM1 and ANGPT2 genes are encoding the angiogenesis-related molecules, which means that BGN is involved in regulating angiogenesis. Also, since biglycan-deficient mice exhibited no embryonic lethality [25], targeting biglycan may not cause severe damage in normal tissue and is perhaps unlikely to cause adverse effects. These data indicated that stromal biglycan may be a clinically relevant therapeutic target in breast cancer.

We observed that biglycan-deficient tumor vessels showed normalized vasculature phenotypes, such as increased pericyte coverage and decreased vessel perfusion, leading to a less hypoxic environment and increased drug delivery. Also, biglycan depletion in stroma suppressed tumor fibrosis, consistent with a previous report [43]. Normalization of the tumor microenvironment has also been theorized to enhance drug delivery and the efficiency of chemotherapies [36]. Together, these results provide a rationale for the combination therapy of stromal biglycan blockade and the conventional chemotherapeutic agent, paclitaxel. Paclitaxel, widely used to treat breast cancer, both kills and activates tumor cells, thereby increasing chemoresistance and metastasis, which markedly limits its clinical application. Here we observed that paclitaxel had a significantly greater effect on tumor growth in biglycan knockout mice compared to WT mice. Therefore, targeting stromal biglycan may enhance the therapeutic potential of chemotherapeutic agents in treating cancers.

We then investigated how inactivation of stromal biglycan induced vascular normalization mechanistically. Pericytes embedded in the basement membrane of blood vessels are important regulators of angiogenesis and vascular stability [44]. Pericytes also promote maturation of blood vessels by stabilizing endothelial cells [45]. ANGPT2, which is largely secreted from activated ECs, is directly angiogenic, and binding of ANGPT2 to its receptor TIE2 on endothelial cells destabilizes vessels by leading to detachment of pericytes, thereby promoting vascular leakiness [46]. Targeting ANGPT2 signaling in tumor vasculature can restore vascular stability and decrease tumor growth and metastasis [47]. It has been shown that ANGPT2 is downstream of biglycan signaling in telomerase-immortalized microvascular endothelial cells [48]. Furthermore, *BGN* was positively associated with *ANGPT2* expression in human breast cancers. We also demonstrated that *Angpt2* mRNA is downregulated in stromal biglycan-deficient tumors. Our results suggest that biglycan destabilizes tumor blood vessels by mediating *Angpt2* expression. However, biglycan itself indirectly induced *Angpt 2* expression in endothelial cells; we propose that TNF-α stimulates tumor angiogenesis via suppression of vascular integrity and increased perfusion as a result of biglycan signaling. TNF-α is a major factor in inflammation and cancer progression [49]. Furthermore, TNF-α promotes angiogenesis in vivo [50] and remodels blood vessels during inflammation [51]. Targeting TNF-α increases mature blood vessels in rheumatoid arthritis synovium [52].

Biglycan acts as a DAMP, and here we provided evidence demonstrating that stromal biglycan promotes TNF-α expression in tumor cells through binding to TLR2 or TLR4, and subsequent activation of NF-κB and ERK signaling pathways. Consistent with previous reports [53], we also found that increased TNF-α expression led to upregulated *Angpt2* mRNA in ECs. These results indicated that stromal biglycan enhances TNF-α expression in tumor cells and that TNF-α subsequently upregulates *Angpt2* expression in ECs, thus destabilizing tumor blood vessels. Furthermore, expression of *Mcr1*, a M2-like macrophage-associated gene, was decreased in tumor-bearing Bgn KO mice (data not shown), which suggests that M2-like macrophages may be altered by stromal biglycan deficiency. M2-like macrophages play a critical role in promoting the formation of an abnormal tumor vascular network and subsequent tumor progression and invasion [54]. Targeting of M2-like macrophages may also potentially lead to the normalization of tumor vasculature [55]. Biglycan has been demonstrated to trigger the synthesis of the macrophage chemoattractants chemokine (C-C motif) ligand CCL2 and CCL5 [56]; thus, it may be possible that biglycan deficiency modulates tumor vasculature through recruitment of macrophages.

As a DAMP, biglycan induces inflammation and fibrogenesis [57]. Biglycan promotes clustering of TLR2/TLR4 with the purinergic P2X7 and P2X4 receptors, which activate NLRP3 [58]. The NLRP3 inflammasome triggers secretion of IL-1β and IL-18, which are involved in renal fibrogenesis [57, 59]. We also observed that biglycan depletion in stroma suppressed tumor fibrosis as well as downregulated collagen I expression. Furthermore, α-SMA + fibroblasts were fewer in biglycan depleted tumors. Biglycan may activate CAFs via upregulating α-SMA expression, thus enhancing fibrosis. Increasing ECM stiffness can enhance cancer progression and metastasis. Biglycan in fibroblasts promotes melanoma invasiveness via increased tissue stiffness, thereby inducing integrin-β1 expression [43], which is supported by our findings. The ECM in tumors can also impair the function of blood vessels via compression [60]. Targeting stromal biglycan reduced desmoplasia and overcame vascular compression. Furthermore, destabilizing tumor vasculature and increasing fibrosis can lead to tumor hypoxia [61]. We have found that biglycan increased HIF1-α and Glut1 expression in tumor cells. It has been reported that biglycan increased the interaction between NF-kB and the HIF-1a promoter, leading to enhanced promoter activity and increased HIF-1a mRNA levels in endothelial cells [39], which is consistent with our findings. HIF1-α is involved in the induction of Glut1 expression [28]. These data indicated that biglycan might be involved in tumor hypoxia response in tumor cells through upregulating HIF1-α and Glut1 expression. Alleviating hypoxia is highly beneficial for improving cancer treatment [61]. However, the detailed mechanisms by which biglycan mediates tumor desmoplasia and hypoxia remain unclear.

Triple negative breast cancer (TNBC) constitutes 15% of all breast cancers and is defined by the lack the expression of hormone receptors and HER2 [62]. Recently, TNBC has been approved to be treated with an immune checkpoint inhibitor (ICI) by the Food and Drug Administration [63]. Here, we provide evidence showing that targeting stromal biglycan alters the tumor microenvironment and enhances intratumoral infiltration of CD8+ T cells in a TNBC mouse model (E0771 breast cancer). However, further analysis is required to elucidate detailed mechanisms of the antitumor immune responses following targeting of biglycan. Furthermore, as biglycan activates the NF-κB signaling pathway, which is one transcriptional activator of PD-L1 [64], we speculate that biglycan may induce PD-L1 expression in tumor cells. Thus, targeting biglycan may achieve superior antitumor effects via multiple parallel pathways.

## Conclusions

Our findings demonstrated that stromal biglycan alters breast cancer microenvironment, and deletion of stromal biglycan enhances the efficacy of chemotherapy treatment. Biglycan may represent a promising candidate to potentiate the efficacy of anticancer therapies in breast cancer.

## Supplementary Information

**Additional file 1:.** Supplementary Figure.

**Additional file 2:.** Supplementary Table.

## Data Availability

The datasets used during the current study are available from the corresponding author on reasonable request.
