# Supplementary material for: Inhibition of stromal biglycan promotes normalization of the tumor microenvironment and enhances chemotherapeutic efficacy
Source: Breast Cancer Res. 2021 May 10;23:51. doi: 10.1186/s13058-021-01423-w (PMC8108358; doi:10.1186/s13058-021-01423-w)
Supplement: Supplementary file 1 — Additional file 1:. Supplementary Figure. [file 13058_2021_1423_MOESM1_ESM.doc]

**Supplementary data**

**Supplementary Fig. 1 Biglycan has no effect on the proliferation of E0771 cells**

The proliferation of E0771 cells treated with recombinant biglycan (100 ng/ml, 1 μg/ml and 5 μg/ml) was performed using an MTS assay after 24 h, 48 h, and 92 h. *p* > 0.05.

**Supplementary Fig. 2 Biglycan expression in E0771 cells and characteristics of isolated TECs derived from E0771 tumors in WT mice**

(A) Biglycan expression was determined by RT-PCR. Skin ECs and TECs derived from A375SM tumors were negative control and positive control of biglycan expression, as previously reported. (B) E0771-TECs were positive for CD31, CD105, and CD144 by RT–PCR. Mouse tumor stromal CD31- cells were also analyzed. TECs were negative for the monocyte marker CD11b and haematopoietic marker CD45. (C) The binding of lectin BS1-B4 and expression of CD31 and CD105 (blue line), and negative expression of CD45 indicated the high purity of isolated TECs. The isotype control is shown as a black line.

**Supplementary Fig. 3 Bgn KO mice show no difference in vascular density of normal mammary gland compared to WT mice**

(A) Representative images of CD31+ blood vessels in normal mammary glands from WT and Bgn KO mice. (B) Quantification of blood vessel density. n=3 mice per group, 3 images per tumor. Scale bar = 100 μm. All data were presented as means ± SD. **p* < 0.05.

**Supplementary Fig. 4 Hif1a and Slc2a1 expression increases in biglycan-stimulated E0771 cells**

(A) *Hif1a* mRNA expression in E0771 cells treated with recombinant biglycan (5 μg/ml) by quantitative RT-PCR. (B) *Slc2a1* mRNA expression in E0771 cells treated with recombinant biglycan (5 μg/ml) by quantitative RT-PCR.

**Supplementary Fig. 5 TNF-ɑ expression increases in biglycan-stimulated RAW cells, and biglycan has no autocrine effect on TNF-ɑ expression**

(A) *Tnfa* mRNA expression in RAW cells treated with recombinant biglycan (5 μg/ml) by quantitative RT-PCR. (B) *Tnfa* mRNA expression in MS1 cells treated with recombinant biglycan (5 μg/ml) by quantitative RT-PCR. (C) *Tnfa* mRNA expression in E0771-TECs transfected with *Bgn* siRNA by quantitative RT-PCR. ****p* < 0.001.

**Supplementary Fig. 6 TNFR1 and TNFR2 expression in MS1 cells.**

Expression of TNFR1 (*Tnfrsf1a*) and TNFR2 (*Tnfrsf1b*) in MS1 cells, dermal ECs, and E0771-TECs by RT-PCR. RAW cells were used as a positive control.

**Supplementary data**

**Supplementary Fig. 1**

**
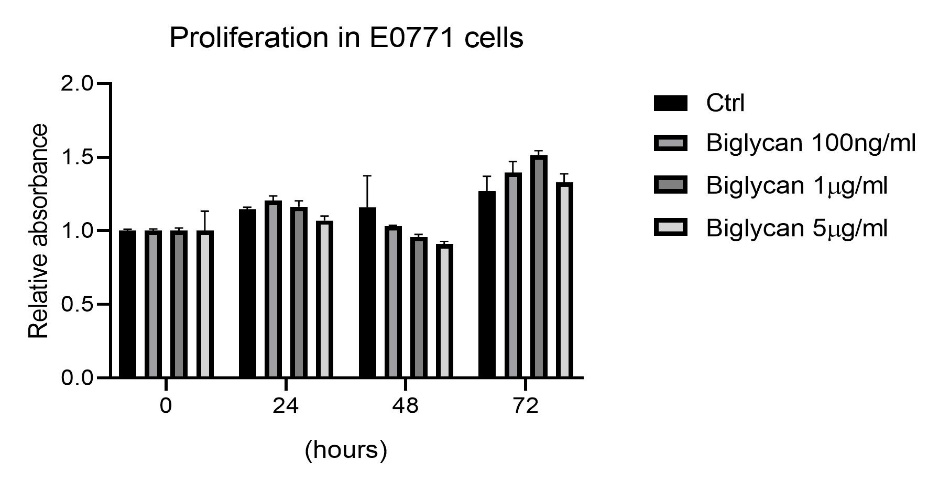
**

**Supplementary Fig. 2**

**
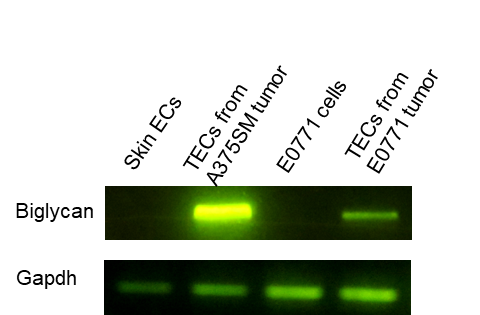
** **
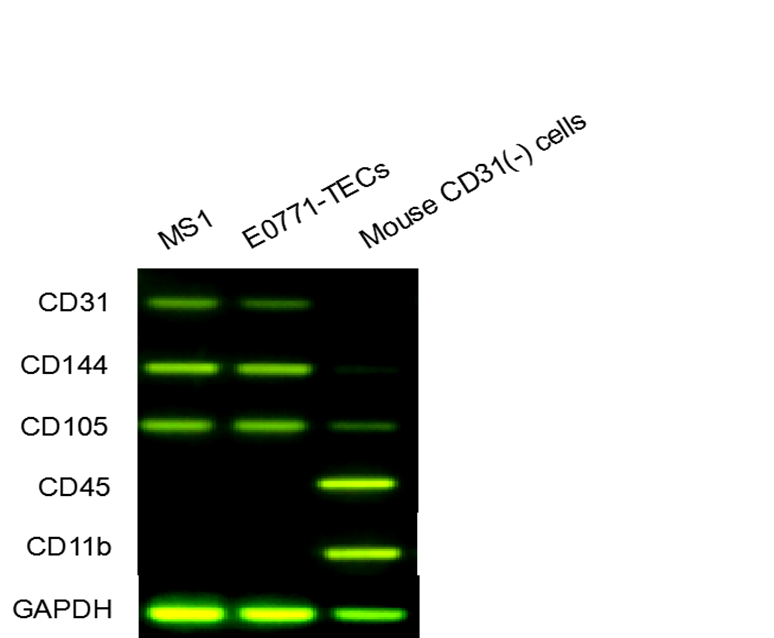
**

**a**

**b**

**
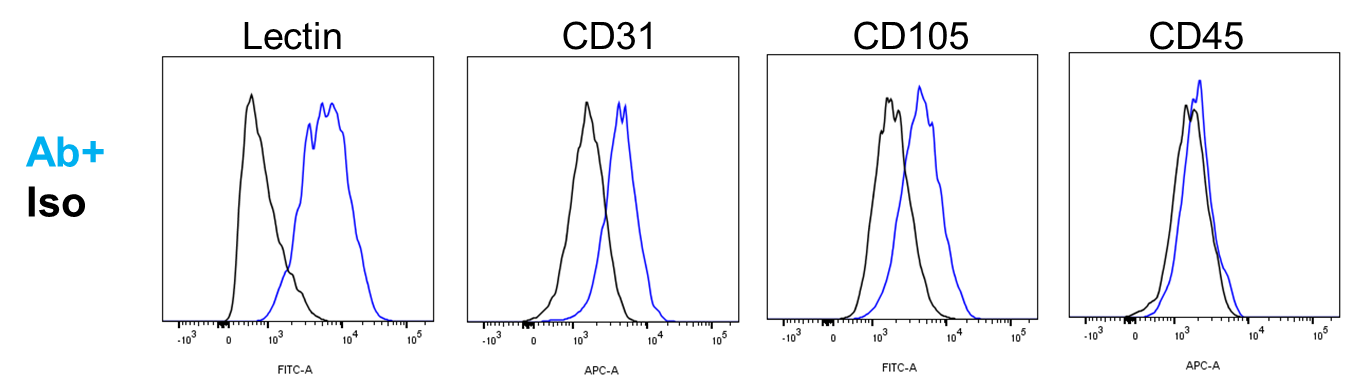
**

**c**

**Supplementary Fig. 3**

**a**

**b**

**
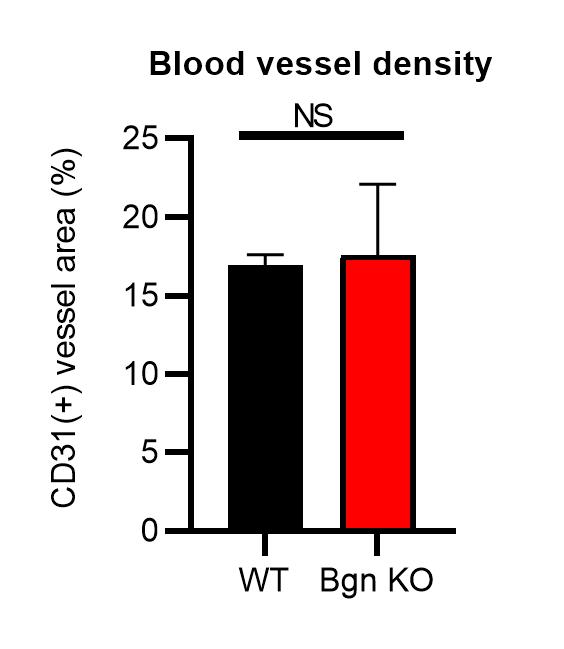

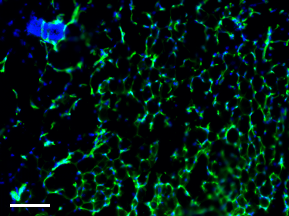

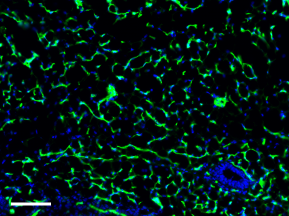
**

**WT**

***Bgn* KO**

**CD31 DAPI**

**Supplementary Fig. 4**

**a**

**b**


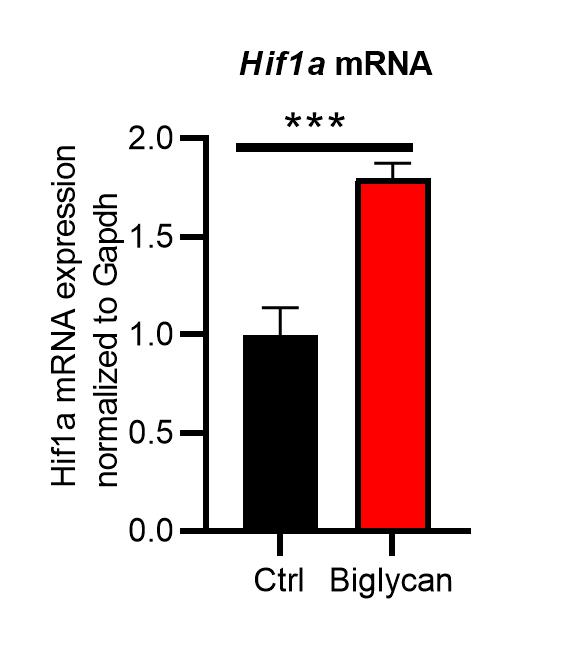

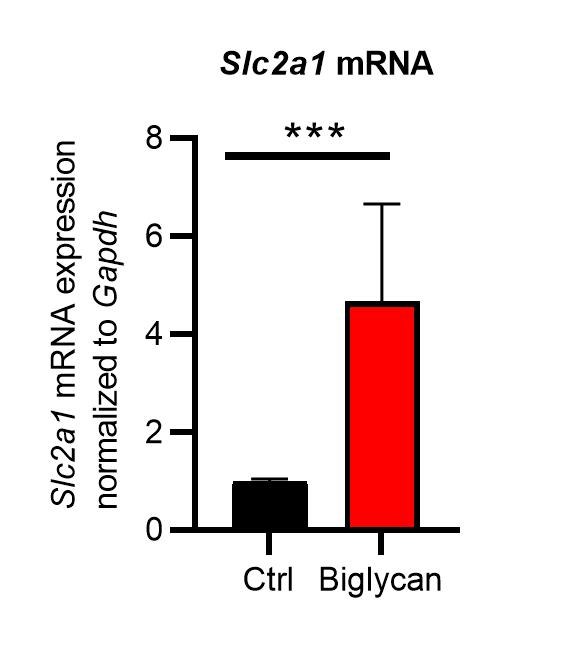


**Supplementary Fig. 5**

**c**

**
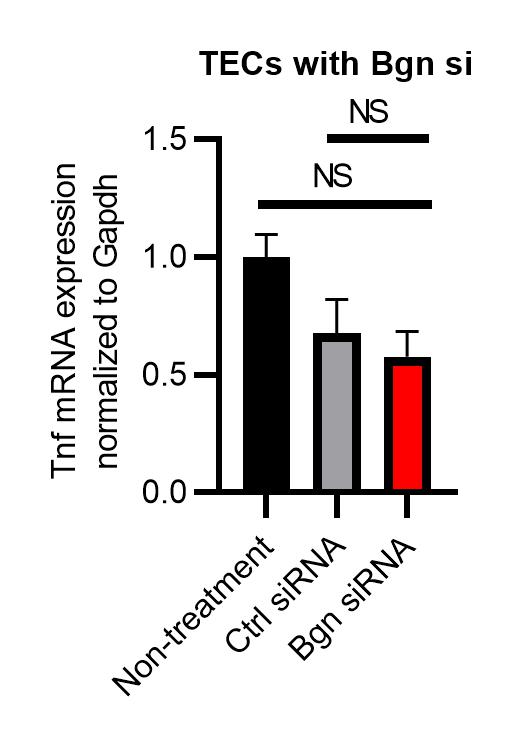

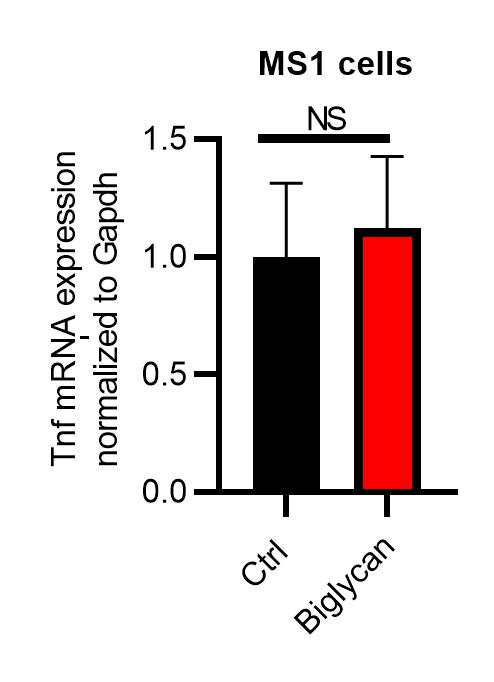

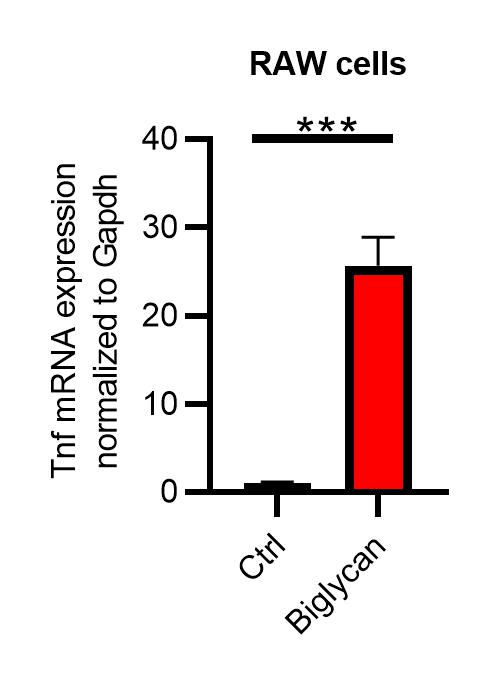
**

**a**

**b**

**Supplementary Fig. 6**

**
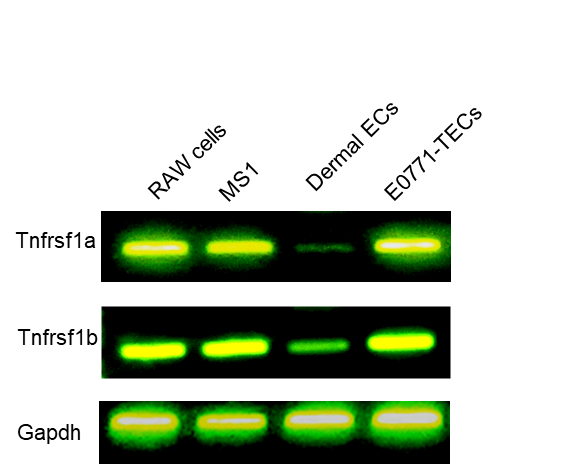
**
