# Supplementary material for: Inhibition of stromal biglycan promotes normalization of the tumor microenvironment and enhances chemotherapeutic efficacy
Source: Breast Cancer Res. 2021 May 10;23:51. doi: 10.1186/s13058-021-01423-w (PMC8108358; doi:10.1186/s13058-021-01423-w)
Supplement: Supplementary file 2 — Additional file 2:. Supplementary Table. [file 13058_2021_1423_MOESM2_ESM.doc]

**Supplementary table S1 List of primers for PCR analysis**

| Genes | Sequences |
| --- | --- |
| *Bgn* | Forward 5'-AACTCACTGCCCCACCACAGCTTC-3' |
|  | Reverse 5'-GCGGTGGCAGTGTGCTCTATCCATC-3' |
| *Angpt2* | Forward 5'-ACTCACCACCAGTGGCATCTAC-3' |
|  | Reverse 5'- TCTCGGTGTTGGATGACTGTCC-3' |
| *Tnf* | Forward 5'-AAGCAAGCAGCCAACCAGGCAG-3' |
|  | Reverse 5'-CGTCGCGGATCATGCTTTCTGTGC-3' |
| *Col1a1* | Forward 5'-GAGCGGAGAGTACTGGATCG-3' |
|  | Reverse 5'-GTTCGGGCTGATGTACCAGT-3' |
| *Cd4* | Forward 5’- CAGGTCTCGCTTCAGTTTGCTG-3’ |
|  | Reverse 5'- GCTGAGCCACTTTCATCACCAC-3’ |
| *Klrb1c* | Forward 5’- TTAGAGTGCCCACAAGACTGGC-3’ |
|  | Reverse 5’- TCAGCTTGACCTTCCTCCCAAG-3’ |
| *Cd27* | Forward 5’- GCTGCAGGCATTGTAACTCTGG-3’ |
|  | Reverse 5’- TCTGTGCCATGAGGTAAGTGGG-3’ |
| *Adgre1* | Forward 5’- TACAAGTGTCTCCCTCGTGCTG-3’ |
|  | Reverse 5’- TTCATCTTGTCCCCTCTGGCTG-3’ |
| *Cd8* | Forward 5’-AGCCCCAGAGACCAGAAGATTG-3’ |
|  | Reverse 5'-CATTTGCAAACACGCTTTCGGC-3’ |
| *Hif1a* | Forward 5’-AAACACTCCTAACTTTTCCCAGCC-3’ |
|  | Reverse 5’-TGGGATATAGGGAGCCAGCATC-3’ |
| *Slc2a1* | Forward 5’-GAGTGACGATCTGAGCTACGGG-3’ |
|  | Reverse 5’-GAACGGACGCGCTGTAACTATG-3’ |
| *Acta2* | Forward 5’-TTCCTTCGTGACTACTGCCGAG-3’ |
|  | Reverse 5’-ATAGGTGGTTTCGTGGATGCCC-3’ |
| *Gapdh* | Forward 5'-CACTGAGCATCTCCCTCACA-3' |
|  | Reverse 5'-GTGGGTGCAGCGAACTTTAT-3' |
| *Actb* | Forward 5'-TTTGCAGCTCCTTCGTTGCCGG-3' |
|  | Reverse 5'-TTTGCACATGCCGGAGCCGTTG-3' |
